# The Fairy Chemical Imidazole-4-carboxamide Inhibits the Expression of Axl, PD-L1, and PD-L2 and Improves Response to Cisplatin in Melanoma

**DOI:** 10.3390/cells11030374

**Published:** 2022-01-22

**Authors:** Chisa Inoue, Taro Yasuma, Corina N. D’Alessandro-Gabazza, Masaaki Toda, Valeria Fridman D’Alessandro, Ryo Inoue, Hajime Fujimoto, Hajime Kobori, Suphachai Tharavecharak, Atsuro Takeshita, Kota Nishihama, Yuko Okano, Jing Wu, Tetsu Kobayashi, Yutaka Yano, Hirokazu Kawagishi, Esteban C. Gabazza

**Affiliations:** 1 Department of Diabetes, Metabolism and Endocrinology, Mie University Faculty and Graduate School of Medicine, Tsu 514-8507, Japan; chisa.i.0417@gmail.com (C.I.); t-yasuma0630@clin.medic.mie-u.ac.jp (T.Y.); johnpaul0114@yahoo.co.jp (A.T.); kn2480@gmail.com (K.N.); yu.higako@gmail.com (Y.O.); yanoyuta@clin.medic.mie-u.ac.jp (Y.Y.); 2 Department of Immunology, Mie University Faculty and Graduate School of Medicine, Tsu 514-8507, Japan; dalessac@clin.medic.mie-u.ac.jp (C.N.D.-G.); t-masa@doc.medic.mie-u.ac.jp (M.T.); immunol@doc.medic.mie-u.ac.jp (V.F.D.); ryo_inoue@ciea.or.jp (R.I.); 3 Central Institute for Experimental Animals, Kawasaki-ku, Kawasaki 210-0821, Japan; 4 Department of Pulmonary and Critical Care Medicine, Mie University Faculty and Graduate School of Medicine, Tsu 514-8507, Japan; genfujimoto1974@yahoo.co.jp (H.F.); kobayashitetsu@hotmail.com (T.K.); 5 Iwade—Research Institute of Mycology Co., Ltd., Tsu 514-0012, Japan; kobori@iwade101.com; 6 Department of Agriculture, Graduate School of Agriculture, University of Miyazaki, Miyazaki 889-2192, Japan; suppp147@gmail.com; 7 Research Institute of Green Science and Technology, Shizuoka University, Shizuoka 422-8529, Japan; wu.jing@shizoka.ac.jp (J.W.); kawagishi.hirokazu@shizuoka.ac.jp (H.K.)

**Keywords:** mushroom, fairy chemicals, cancer, drug resistance, Axl, immune checkpoint molecules

## Abstract

The leading cause of death worldwide is cancer. Many reports have proved the beneficial effect of mushrooms in cancer. However, the precise mechanism is not completely clear. In the present study, we focused on the medicinal properties of biomolecules released by fairy ring-forming mushrooms. Fairy chemicals generally stimulate or inhibit the growth of surrounding vegetation. In the present study, we evaluated whether fairy chemicals (2-azahypoxanthine, 2-aza-8-oxohypoxanthine, and imidazole-4-carboxamide) exert anticancer activity by decreasing the expression of Axl and immune checkpoint molecules in melanoma cells. We used B16F10 melanoma cell lines and a melanoma xenograft model in the experiments. Treatment of melanoma xenograft with cisplatin combined with imidazole-4-carboxamide significantly decreased the tumor volume compared to untreated mice or mice treated cisplatin alone. In addition, mice treated with cisplatin and imidazole-4-carboxamide showed increased peritumoral infiltration of T cells compared to mice treated with cisplatin alone. In vitro studies showed that all fairy chemicals, including imidazole-4-carboxamide, inhibit the expression of immune checkpoint molecules and Axl compared to controls. Imidazole-4-carboxamide also significantly blocks the cisplatin-induced upregulation of PD-L1. These observations point to the fairy chemical imidazole-4-carboxamide as a promising coadjuvant therapy with cisplatin in patients with cancer.

## 1. Introduction

Cancer is the most frequent cause of death worldwide, according to the World Health Organization [1]. There were about 10 million cancer-related deaths globally in 2020, and in Japan, approximately 70,000 people are dying from cancer every year [1]. Therefore, there is a compelling need to develop new and more effective therapeutic modalities against malignant tumors. The development of small-molecule inhibitors of receptor tyrosine kinase activation caused by cancer driver genes and immunotherapy targeting immune checkpoint molecules that stimulate the host immune system to kill malignant cells are some of the greatest achievements in the field of cancer therapy [2,3,4,5,6]. In particular, the treatment with monoclonal antibodies against the immune checkpoint molecules programmed death-ligand 1 (PD-L1) and programmed cell death protein 1 (PD-1) has improved the survival of patients with malignant tumors, including advanced melanoma, non-small cell lung cancer, and renal cell carcinoma [6,7]. However, acquired resistance develops in most patients receiving these new therapies, leading to cancer progression and fatal outcomes [8,9]. An important mechanism of acquired resistance to anticancer treatment is the overexpression of the Axl receptor tyrosine kinase [10,11,12]. Increased expression and activation of Axl contribute to primary and acquired drug resistance by stimulating mesenchymal differentiation and survival of cancer cells and suppressing the host immune response [13,14,15,16]. Furthermore, Axl activation can also promote the expression of immune checkpoint molecules and tumor growth [17]. These observations point to Axl as an important target for overcoming cancer drug resistance.

Mushrooms, along with rusts, molds, yeasts, and mildews, belong to the kingdom Fungi. There are nearly 12,000 species of mushrooms on earth [18,19,20]. They have long been used as food and traditional medicine [18,21]. Mushrooms are a rich source of nutrients, including essential amino acids, polysaccharides, vitamins, and dietary fibers. Several chronic diseases, including cancer, have been empirically treated with mushrooms, and recent studies have documented their pharmacological properties [22]. However, the molecular component and the precise mechanism responsible for their beneficial health effects are unclear. Previous studies suggested that fruiting body extracts from mushrooms, including *Leucopaxillus giganteus*, and *Pleurocybella porrigens* may stimulate host immunity by suppressing the expression of Axl, PD-L1, and PD-L2 in the non-small cell lung cancer cell lines A549 and H3255 cells [23,24]. Apart from decreasing the expression of Axl and immune checkpoint molecules in the non-small cell lung cancer cell lines A549, the subcritical water extracts of *Agaricus blazei* Murril’s mycelium also stimulate the maturation of dendritic cells [25]. In the present study, we focused on the medicinal properties of fairy chemicals produced by mushrooms that form fairy rings. Fairy rings are colonies of soil-growing fungi that form annular or semicircular structures [26,27,28,29]. Apart from the circular alignment, the fairy ring-forming mushrooms are notorious on the ground because they generally stimulate or inhibit the growth of surrounding vegetation, such as lawns (playing fields or urban), mown hay fields, or pastures. We recently demonstrated using the fungus *Lepista sordida* (Komurasakishimeji in Japanese) that 2-azahypoxanthine (AHX) and its metabolite 2-aza-8-oxohypoxanthine (AOH) are the fairy chemicals responsible for the growth-stimulating activity and imidazole-4-carboxamide (ICA) for the suppressive activity [29,30,31,32]. We also found that the fairy chemicals regulate the growth of various plants, including rice, wheat, corn, potato, tomato, lettuce, and asparagus, suggesting their potential application in agriculture [33]. In addition, AHX suppresses retinal angiogenesis as a hypoxia-inducible factor inhibitor [34]. Based on these regulatory activities and the well-recognized pharmacological properties of mushrooms, in the present study, we hypothesized that fairy chemicals promote anticancer activity by decreasing the expression of Axl and immune checkpoint molecules in cancer cells.

## 2. Materials and Methods

### 2.1. Animals

Female C57BL/6J wild-type (body weight: 21–24; age: 10–11 weeks) mice were provided from Nihon SLC (Hamamatsu, Tokyo, Japan). The mice were maintained in a specific pathogen-free environment at a temperature of 21 °C and under a 12-h light/dark cycle in the animal facility of Mie University. The cage of the mice contains wood-wool nesting material, and mice had access to water and food ad libitum.

### 2.2. Ethical Statement

The Committee for Animal Investigation of Mie University approved the experimental protocols (approval No.: 2020-10; approval date: 14 November 2020). We performed all experimental procedures following internationally approved laboratory animal care principles published by the National Institute of Health (https://olaw.nih.gov/ (accessed on 6 June 2021). In addition, the research followed the ARRIVE Guidelines for animal investigation, and variables were measured blindly in the treatment groups.

### 2.3. Mouse Model of Melanoma and Treatment Schedule

ICA (molecular weight, 111.1 Daltons) significantly inhibited PD-L1 expression in B16F10 melanoma cells at a concentration of 100 µg/mL (0.9 mM). We used this concentration as a reference to estimate the approximate ICA dose required to induce inhibitory activity in our in vivo cancer model. The following molarity equation and the molarity calculator software provided by GraphPad (https://www.graphpad.com/quickcalcs/molarityform/ (accessed on 8 August 2021)) were used to calculate the ICA mass (g): M = C × V × MW. Where M is the mass of ICA in gram, C is the in vitro concentration of ICA that inhibits PD-L-1 expression in vitro expressed in mol/L, V is the bodyweight of a mouse expressed in volume (1 g = 0.001 L), and MW is the molecular weight of ICA in Daltons. The calculator software provides the following result:

ICA (g) = 0.0009 mol/L × 0.024 L × 111.1 Daltons = 0.002399 g = 2.399 mg

Mice were separated into four treatment groups: an ICA/cisplatin group treated with both ICA and cisplatin; a cisplatin group treated only with cisplatin; an ICA group treated only with ICA and an untreated group. ICA (2.5 mg per mouse) was administered by intraperitoneal injection three times a week for three weeks and cisplatin (2.5 mg/kg mouse weight) also by intraperitoneal route once a week for three weeks. An allograft melanoma model was induced by intradermal injection of a suspension of 1 × 10^5^ B16F10 melanoma cells in 100 μL of Dulbecco’s Modified Eagle Medium (DMEM) into the right flank of mice under profound anesthesia with isoflurane [35]. The tumor volume was monitored by CT scan during the entire experiment, and it was calculated using the following formula: V = L × W2/2, where V is tumor volume, L length, and W width.

### 2.4. Apoptosis and MTT Assay

B16F10 cell lines (4 × 10^5^ cells/well) were seeded into 12-well plates, cultured up to sub-confluency and then treated with 20 µg/mL of ICA + saline, 20 µM of cisplatin + saline, or with a combination ICA and cisplatin for 24 h at 37 °C before evaluating apoptosis by flow cytometry. Cells culture in the presence of saline was used as controls. Apoptosis was assessed using a flow cytometer (FACScan, BD Biosciences, Oxford, UK) after staining with fluorescein-labeled annexin V and propidium iodide (FITC Annexin V Apoptosis Detection Kit with PI, Biolegend, San Diego, CA, USA). The 3-(4,5-dimethylthiazol-2-yl)-2,5-diphenyl-2H-tetrazolium bromide (MTT) assay was performed using a commercial kit (CellTiter 96 Aqueous one Solution Cell Proliferation Assay) from Promega (Madison, WI, USA).

### 2.5. CT Examination

CT of the lungs was performed with a micro-CT Latheta LCT-200 purchased from Hitachi Aloka Medical (Tokyo, Japan). After anesthesia with isoflurane inhalation, mice were placed prone for data acquisition [36].

### 2.6. Euthanasia and Collection of Samples

On day 18, mice were sacrificed by an overdose of isoflurane inhalation. Blood was drawn by cardiac puncture and transferred to heparinized tubes. After centrifugation at 10,000 rpm for 3 min at 4 °C, plasma was collected in tubes and stored at −80 °C until analysis. The tumor xenograft was incised in a block with the surrounding skin, embedded in paraffin, and prepared for immunohistochemistry.

### 2.7. Immunohistochemistry

Immunostaining of xenograft tissue with CD3^+^ or CD8^+^ was performed at MorphoTechnology Corporation (Sapporo, Hokkaido, Japan) using anti-mouse CD3^+^ (GeneTex, Irvine, CA, USA) or anti-mouse CD8α^+^ (Cell Signaling, Danvers, MA, USA) monoclonal antibodies, Histofine^®^Simple Stain MAX PO (R) and 3,3′-diaminobenzidine.

### 2.8. Preparation of AHX, AOH, and ICA

AHX was synthesized from 5-aminoimidazole-4-carboxamide and converted to AOH by resting cells of *Burkholderia contaminans* as described previously. Imidazole-4-carboxamide was synthesized as previously described [31,37,38].

### 2.9. Cell Lines and Reagents

The human lung adenocarcinoma cell line A549 and the mouse melanoma cell line B16F10 were from the American Type Culture Collection (Manassas, VA, USA). DMEM was from Sigma-Aldrich (Saint Louis, MO, USA), and the fetal bovine serum (FBS) was from Bio Whittaker (Walkersville, MD, USA). L-glutamine, penicillin, and streptomycin were purchased from Invitrogen (Carlsbad, CA, USA).

### 2.10. Cell Culture

The A549 and B16F10 cell lines were cultured in DMEM containing 10% FBS, L-glutamine, and sodium pyruvate in a 5% CO_2_/95% air atmosphere at 37 °C.

### 2.11. Cell Stimulation

The cell lines were seeded on 12-well plates, cultured up to sub-confluency, serum-starved for 12 h, and then stimulated with 20 µg/mL of AHX, AOH, or ICA for 24 h at 37 °C before assessing the cells by reverse transcriptase-polymerase chain reaction (RT-PCR), flow cytometry, or Western blotting. Supplementary Table S1 described the source of antibodies. Cells cultured similarly in the presence of an equal amount of saline (SAL) were the negative controls, and cells stimulated with the fruiting body alkaline extract from *Agaricus blazei* Murrill were the positive controls [25].

In a separate experiment, B16F10 cells were cultured similarly and treated with varying concentrations of ICA, positive or negative control. In addition, to evaluate the suppressive activity of ICA on cisplatin-induced upregulation of PD-L1, B16F10 cells were cultured in the presence of both cisplatin (10 µM) and ICA (100 µg/mL), and the same concentration of cisplatin or ICA alone for 24 h and then the expression of PD-L1 was evaluated by RT-PCR or flow cytometry.

### 2.12. Evaluation of Gene Expression

The total RNA was extracted from the cell lines using Sepasol RNA-I Super G reagent (Nacalai Tesque Inc., Kyoto, Japan), and the cDNA was prepared using 2 μg of total RNA oligo-dT primers and Reverse Transcriptase (Toyobo Life Science Department, Osaka, Japan). The primers to carry out RT-PCR were previously reported [23]. RT-PCR was performed under the following conditions: cycles between 26 to 35, denaturation at 94 °C for 30 s, annealing at 65 °C for 30 s, elongation at 72 °C for 1 min, and extension at 72 °C for 5 min. The mRNA expression was normalized by the glyceraldehyde 3-phosphate dehydrogenase (GAPDH) mRNA expression.

### 2.13. Western Blotting

Analysis of protein expression by western blotting was performed by classical methods. Briefly, cultured cells were washed twice with ice-cold phosphate-buffered saline and then treated with lysis buffer containing a cocktail of protease/phosphatase inhibitors. The lysate was then centrifuged, and the protein concentration was measured using the Pierce BCA protein assay kit (Thermo Fisher Scientific Incorporation, Waltham, MA, USA). Samples with an equal protein concentration were mixed with Laemmli sample buffer and run in a sodium dodecyl sulfate-polyacrylamide gel electrophoresis. After protein transfer to a nitrocellulose membrane followed by appropriate washing, the membrane was treated with antibodies against PD-L1, PD-L2, Axl, and βactin. Supplementary Table S1 described the source of antibodies. The intensity of the blots on the membrane was quantified using the public domain NIH ImageJ program (wayne@codon.nih.gov; Wayne Rasband, NIH, Research Service Branch) [39].

### 2.14. Flow Cytometry Analysis

After staining with specific antibodies, PD-L1, PD-L2, and Axl expression was evaluated by flow cytometry and CELL Quest software (FACScan, BD Biosciences, Oxford, UK).

### 2.15. Statistical Analysis

The results are expressed as the mean ± standard deviation of the means (S.D.) unless otherwise specified. The statistical difference between three or more variables was calculated by ANOVA with posthoc analysis using Fisher’s predicted least significant difference test. *p*-value < 0.05 was considered statistically significant. We used GraphPad Prism vs. 7 (GraphPad Software, Inc., San Diego, CA, USA) to perform the statistical analysis.

## 3. Results

### 3.1. ICA Decreases PD-L1 Expression Upregulation Induced by Cisplatin in B16F10 Cells

**Figure 1:**
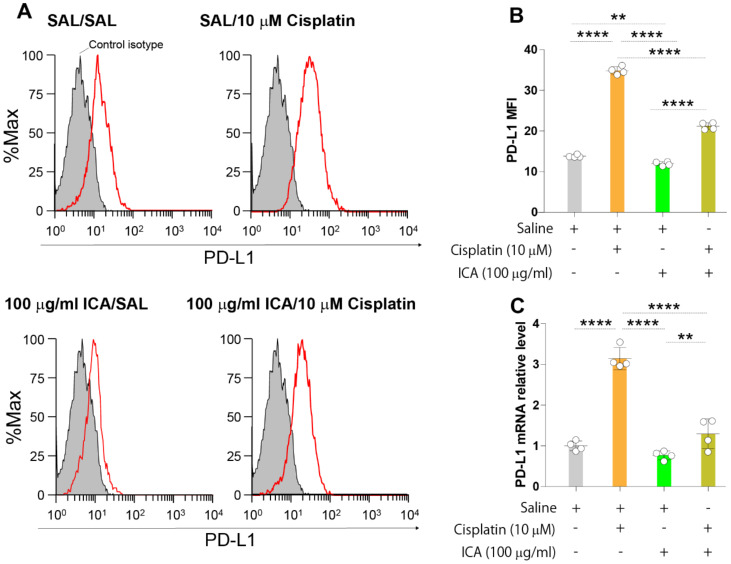
A high dose of ICA decreases cisplatin-induced PD-L1 expression in B16F10 cells. (**A**,**B**): B16F10 cells were cultured in the presence of saline, ICA alone (100 µg/mL), or cisplatin alone (10 µM) and in the presence of the same concentrations of both cisplatin and ICA for 24 h, and the surface protein expression of PD-L1 were compared between the different treatment groups by flow cytometry. (**C**) B16F10 cells were cultured in the presence of saline, ICA alone (100 µg/mL), or cisplatin alone (10 µM) and in the presence of the same concentrations of both cisplatin and ICA for 24 h and the mRNA expression of PD-L1 was evaluated by RT-PCR. Data are expressed as the mean ± S.D. *n* = 4 in each treatment group. Statistical analysis was performed by ANOVA with Fisher’s predicted least significant difference test. PD-L1, programmed death-ligand 1; PD-L2, programmed death-ligand 2; ICA, imidazole-4-carboxamide; ns, not significant. ** *p* < 0.01; **** *p* < 0.0001.

Previous studies have shown that cisplatin upregulates the expression of PD-L1 in cancer cells [40,41,42]. Therefore, we evaluated whether the fairy chemical ICA can decrease the expression of PD-L1 induced by cisplatin in B16F10 melanoma cells. Cells were cultured in the presence of saline, ICA, or cisplatin alone and in the presence of both cisplatin and ICA for 24 h, and the surface protein expression and mRNA expression of PD-L1 were compared between the different treatment groups (Figure 1A–C). Cisplatin significantly increased the protein expression and the relative mRNA expression of PD-L1 compared to saline. ICA significantly reduced the cisplatin-mediated expression of cell surface protein and mRNA of PD-L1 compared to controls (Figure 1A–C). These results suggest that ICA improves the antitumor activity of cisplatin in melanoma.

### 3.2. The Fairy Chemical ICA Inhibits Tumor Growth in a Mouse Xenograft Melanoma Model

**Figure 2:**
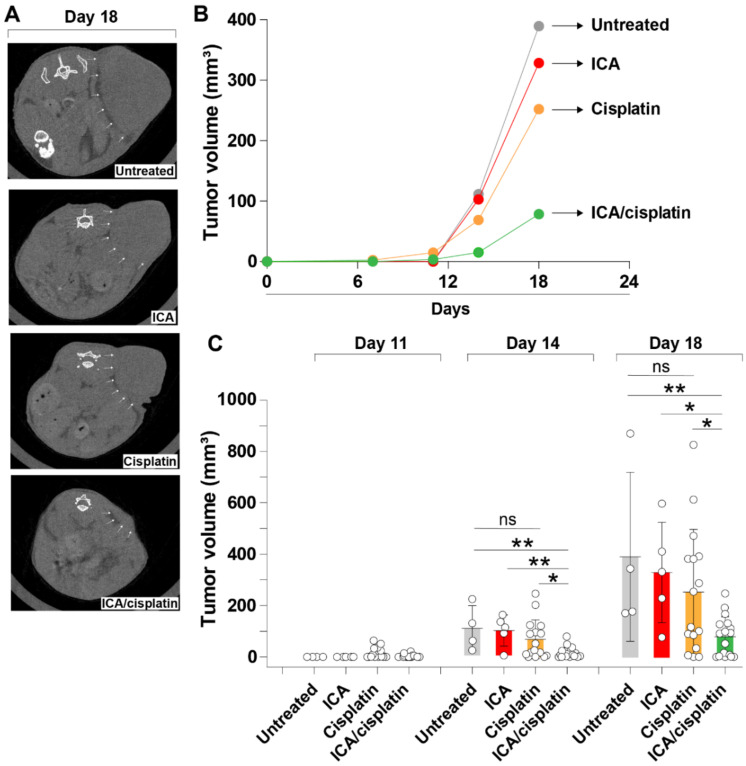
ICA inhibits tumor growth in a mouse xenograft melanoma model. (**A**): Mice received intradermal injections of a suspension of (1 × 10^5^) B16F10 cells and were treated with ICA alone (2.5 mg/mouse; *n* = 5; ICA group), cisplatin alone (2.5 mg/kg mouse weight; *n* = 16; cisplatin group), or the same dose of a combination of ICA and cisplatin (*n* = 16; ICA/cisplatin group). A mouse group (*n* = 4; untreated group) received no treatment. The volume of the xenograft tumor was measured by CT scan. Arrows indicate the tumor region. (**B**,**C**): The volume of the xenograft tumor was followed up for several days. Data are expressed as the mean ± S.D. Statistical analysis was performed by ANOVA with Fisher’s predicted least significant difference test. ICA, imidazole-4-carboxamide; ns, not significant. * *p* < 0.05; ** *p* < 0.01.

Mushrooms exert anticancer activity and improve the cytotoxic activity of cisplatin on cancer cells [43,44,45]. In addition, ICA has been reported to suppress the growth of vegetation surrounding the fairy tales formed by mushrooms [28]. Based on these previous studies and the observation that ICA significantly inhibits the expression of PD-L1 in B16F10 melanoma cells, we hypothesized that systemic administration of ICA and cisplatin would inhibit tumor growth in a mouse xenograft melanoma model. In the experiments, mice received intradermal injections of B16F10 cell suspensions and were treated with ICA alone (ICA group), cisplatin alone (cisplatin group), or ICA plus cisplatin (ICA/cisplatin group). A mouse group (untreated group) received no treatment. The volume of the tumor xenograft was longitudinally measured by CT scan and compared between the different treatment groups. The ICA/cisplatin group showed a significantly decreased volume of the xenograft tumors compared to the untreated group, ICA, and cisplatin groups on days 14 and 18 after melanoma cell inoculation (Figure 2A–C). There was no significant difference in tumor volume between untreated and cisplatin groups or between untreated and ICA groups (Figure 2A–C). These findings suggest ICA improved the cytotoxic activity of cisplatin against melanomas cells.

### 3.3. ICA Potentiates Cisplatin Activity

Based on the potentiating activity of ICA on the cytotoxic activity of cisplatin, we evaluated whether the results can be recapitulated in vitro. B16F10 melanoma cells cultured in the presence of saline, ICA plus saline, cisplatin plus saline, or ICA plus cisplatin and apoptosis was evaluated by flow cytometry. Cells treated with cisplatin + saline showed significantly increased apoptosis compared to saline controls, and cells treated with a combination of ICA and cisplatin showed significantly increased apoptosis compared to cells treated with cisplatin plus saline. Consistent with these findings, ICA significantly improved the IC_50_ of cisplatin in the MTT assay (Supplementary Figure S1A–C).

### 3.4. Enhanced Infiltration of CD8^+^ Cells in Mice Treated with ICA and Cisplatin

**Figure 3:**
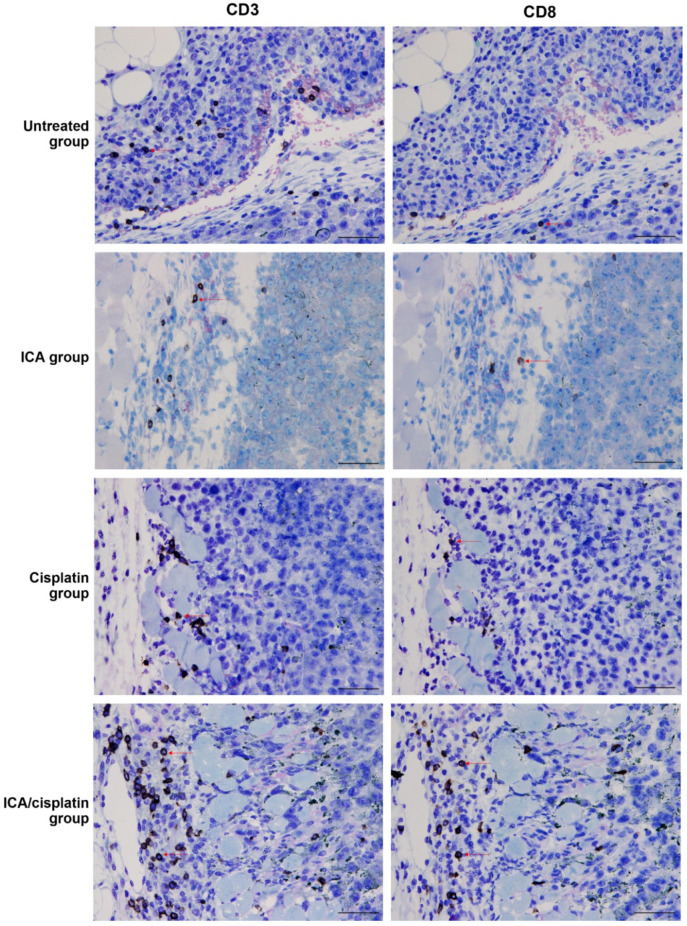
Enhanced infiltration of CD3^+^ and CD8^+^ T cells in mice treated with ICA and cisplatin. Immunostaining of CD3^+^ and CD8^+^ T cells was performed using paraffin-embedded xenograft samples described under Methods. Scale bars indicate 50 µm. Red arrows show CD3^+^ and CD8^+^ T cells. ICA, imidazole-4-carboxamide.

Previous studies have associated the beneficial effects of mushrooms in cancer with increased immune response activation [43,44]. We then hypothesized that treatment with ICA increases the intratumoral infiltration of T cells. To interrogate this hypothesis, we performed immunostaining of CD3+ and CD8+ T cells in melanoma tissues from each treatment group. Immunostaining of CD3^+^ and CD8^+^ T cells was performed using paraffin-embedded xenograft samples (Figure 3). Enhanced infiltration of CD3^+^ and CD8^+^ T cells was observed in the ICA/cisplatin group compared to other treatment groups. These results indicate that systemic administration of ICA increases the T cell response against melanoma cells.

### 3.5. Fairy Chemicals Inhibit the mRNA Relative Expression of Axl, PD-L1, and PD-L2

**Figure 4:**
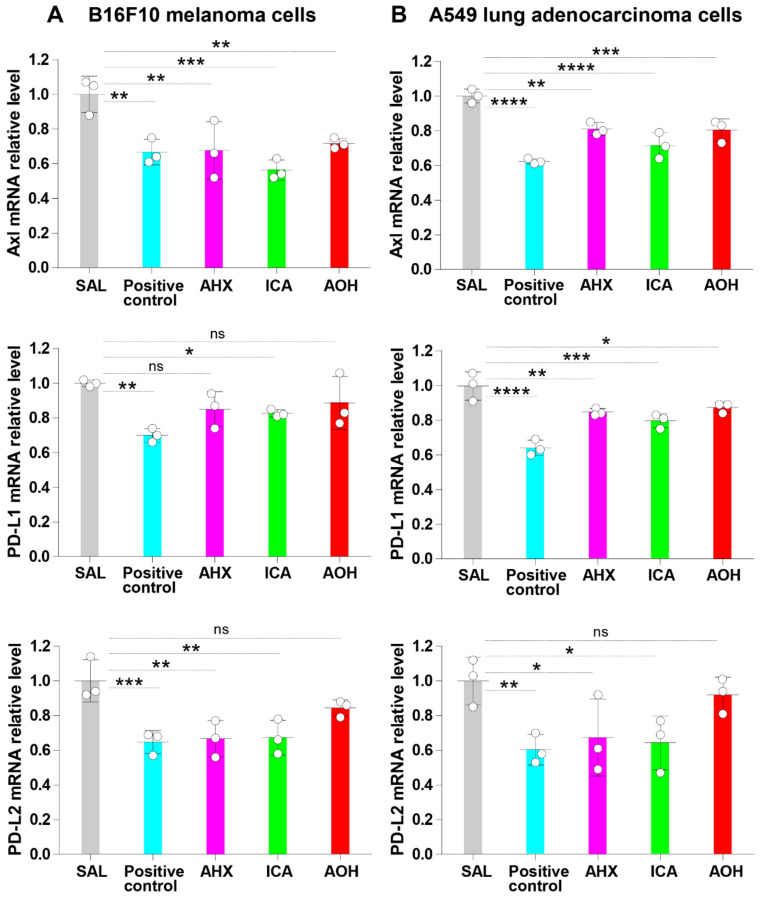
Fairy chemicals inhibit the mRNA relation expression of Axl, PD-L1, and PD-L2. (**A**,**B**): B16F10 and A549 cells (2 × 10^5^ cells/well) were cultured in 12-well plates in the presence of 20 µg/mL of AHX, ICA, AOH, or controls for 24 h, and the expression of Axl and checkpoint molecules was evaluated by RT-PCR. Cells cultured in the presence of saline (SAL) were used as a negative control, and cells cultured in the presence of extract from *Agaricus blazei* Murril (20 µg/mL) were used as positive controls. Data are expressed as the mean ± S.D. *n* = 3 in each treatment group. Statistical analysis was performed by ANOVA with Fisher’s predicted least significant difference test. PD-L1 programmed death-ligand 1; PD-L2 programmed death-ligand 2; AHX, 2-azahypoxanthine; AOH, 2-aza-8-oxohypoxanthine; ICA, imidazole-4-carboxamide; ns, not significant. * *p* < 0.05; ** *p* < 0.01; *** *p* < 0.001; **** *p* < 0.0001.

Reduced expression of Axl, PD-L1 and PD-L2 in cancer cells is linked to the increased antitumor activity of the host immune response [46]. Therefore, we evaluated whether fairy chemicals can suppress the expression of Axl and immune checkpoint molecules in B16F10 melanoma cells. B16F10 cells were cultured in 12-well plates in the presence of the fairy chemicals AHX, ICA, AOH, and controls for 24 h, and the expression of Axl and checkpoint molecules was evaluated by RT-PCR. ICA stimulation of B16F10 cells significantly reduced the relative mRNA expression of Axl, PD-L1, and PD-L2 compared to untreated cells. AHX significantly reduced the relative mRNA expression of Axl and PD-L2 but not that of PD-L1 in B16F10 melanoma cells. AOH significantly reduced the relative mRNA expression of Axl, but not that of PD-L1 or PD-L2 in B16F10 cells (Figure 4A).

Mushroom’s extracts have been shown to inhibit the expression of Axl and immune checkpoint molecules in the lung adenocarcinoma A549 cell lines [25]. Therefore, to validate our findings, we evaluated whether the fairy chemicals can also inhibit the expression of Axl, PD-L1, and PD-L2 in A549 cells. A549 cells stimulated with AHX and ICA showed significantly decreased relative mRNA expression of Axl, PD-L1, and PD-L2 compared to untreated cells. In addition, treatment with AOH significantly reduced the relative mRNA expression of Axl and PD-L1 compared to saline control. The relative mRNA expression of PD-L2 was also low in A549 cells stimulated with AOH, although the decrease was not statistically significant (Figure 4B). These results indicate the inhibitory activity of ICA on mRNA expression of Axl and immune checkpoint molecules in cancer cells.

### 3.6. Inhibition of Axl, PD-L1, and PD-L2 Protein Expression by Fairy Chemicals in Melanoma Cells

**Figure 5:**
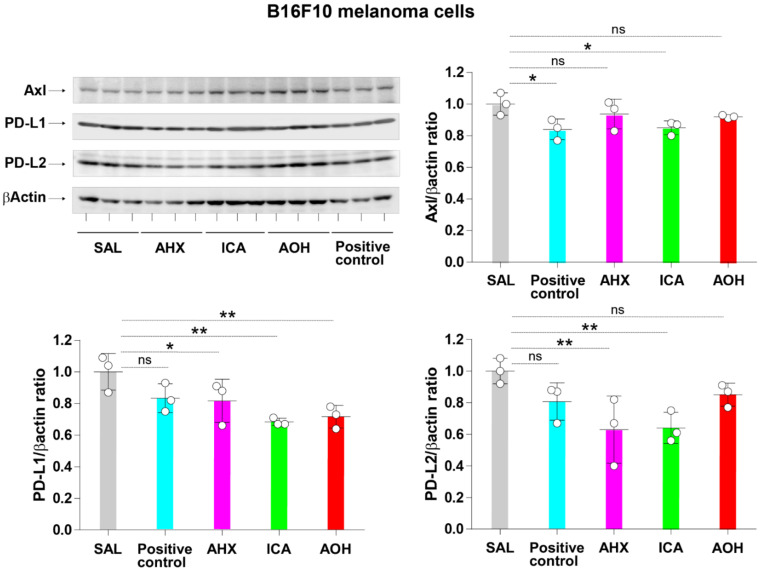
Inhibition of Axl, PD-L1, and PD-L2 protein expression by fairy chemicals in melanoma cells. B16F10 cell lines (2 × 10^5^ cells/well) were cultured in the presence of 20 µg/mL of each fairy chemical or positive control for 24 h, treated with lysis buffer to extract protein, and performed Western blotting. Data are expressed as the mean ± S.D. *n* = 3 in each treatment group. Statistical analysis was performed by ANOVA with Fisher’s predicted least significant difference test. PD-L1 programmed death-ligand 1; PD-L2 programmed death-ligand 2; AHX, 2-azahypoxanthine; AOH, 2-aza-8-oxohypoxanthine; ICA, imidazole-4-carboxamide; SAL, saline; ns, not significant. * *p* < 0.05; ** *p* < 0.01.

Inhibition of the protein expression of Axl and immune checkpoint molecules was evaluated by Western blotting and flow cytometry. B16F10 cell lines were cultured in the presence of each fairy chemical for 24 h, treated with lysis buffer to extract protein, and perform Western blotting. AHX significantly inhibited the protein expression of PD-L1 and PD-L2, ICA significantly inhibited the protein expression of Axl, PD-L1, and PD-L2, and AOH significantly inhibited the protein expression of PD-L1 compared to saline (Figure 5).

### 3.7. Significant Inhibition of the Surface Protein Expression of Axl and Immune Checkpoint Molecules by Fairy Chemicals in Melanoma Cells

**Figure 6:**
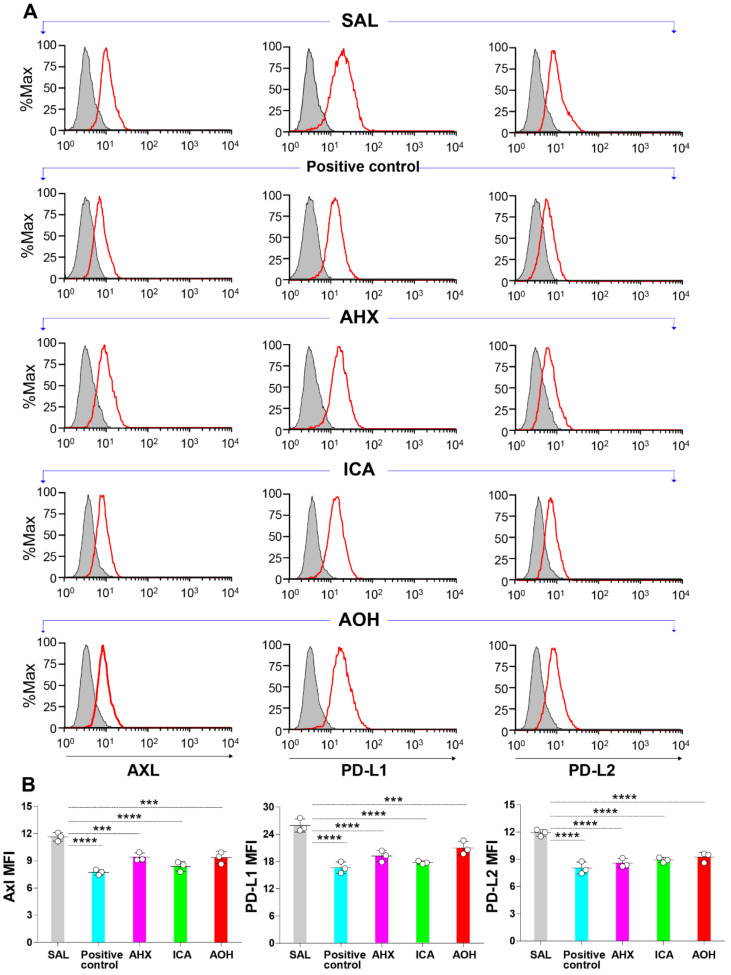
Inhibition of the surface protein expression of Axl and immune checkpoint molecules by fairy chemicals in melanoma cells. (**A**,**B**): B16F10 cell lines (2 × 10^5^ cells/well) were cultured in the presence of 20 µg/mL of each fairy chemical or positive control for 24 h, and then the surface expression of Axl, PD-L1, and PD-L2 was evaluated by flow cytometry. Data are expressed as the mean ± S.D. *n* = 3 in each treatment group. Statistical analysis was performed by ANOVA with Fisher’s predicted least significant difference test. PD-L1 programmed death-ligand 1; PD-L2 programmed death-ligand 2; AHX, 2-azahypoxanthine; AOH, 2-aza-8-oxohypoxanthine; ICA, imidazole-4-carboxamide; SAL, saline; ns, not significant. *** *p* < 0.001; **** *p* < 0.0001.

B16F10 cell lines were cultured in the presence of each fairy chemical for 24 h, and then the surface expression of Axl, PD-L1 and PD-L2 was evaluated by flow cytometry. Compared to cells treated with saline alone, cells treated with AHX, ICA, or AOH showed significantly reduced expression of Axl, PD-L1, and PD-L2 (Figure 6A,B). These observations indicate the inhibitory activity of ICA on protein expression of Axl and immune checkpoint molecules in cancer cells.

### 3.8. ICA Dose-Dependently Downregulates the Expression of Axl and Immune Checkpoint Molecules

**Figure 7:**
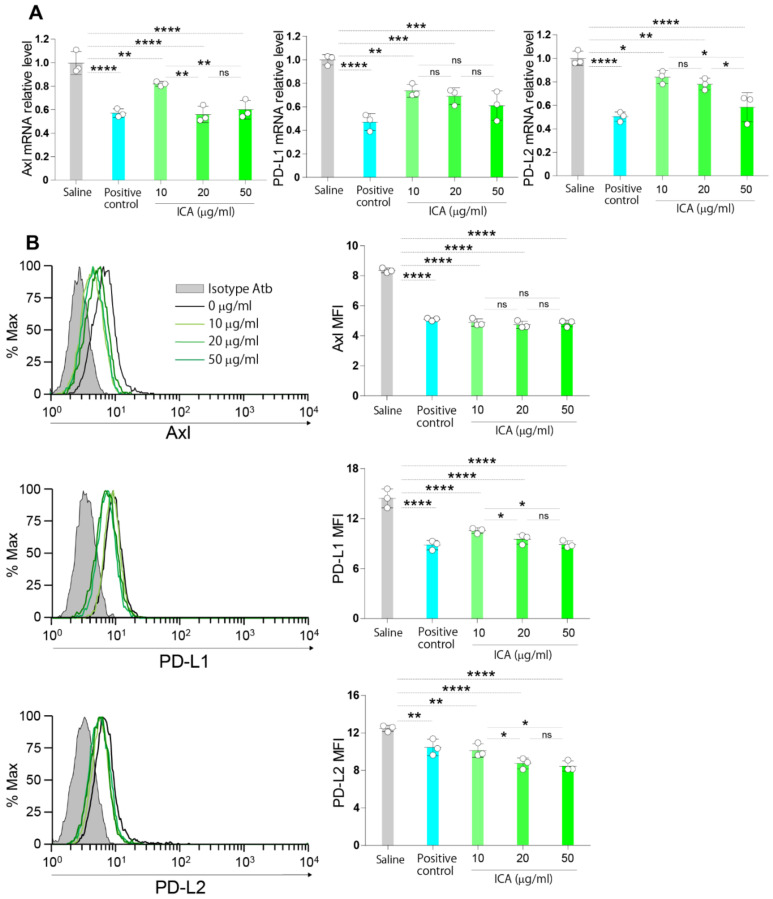
ICA dose-dependently downregulates the expression of Axl and immune checkpoint molecules. (**A**,**B**): B16F10 cell lines were cultured in the presence of varying concentrations (10, 20, 50 µg/mL) of ICA or positive control (20 µg/mL) for 24 h, and the expression of Axl, PD-L1 and PD-L2 was assessed by RT-PCR and flow cytometry. Saline was used as a negative control. Data are expressed as the mean ± S.D. *n* = 3 in each treatment group. Statistical analysis was performed by ANOVA with Fisher’s predicted least significant difference test. PD-L1, programmed death-ligand 1; PD-L2, programmed death-ligand 2; ICA, imidazole-4-carboxamide; ns, not significant. * *p* < 0.05; ** *p* < 0.01; *** *p* < 0.001; **** *p* < 0.0001.

We then focused on the inhibitory activity of ICA and evaluated its dose-dependent effect on the expression of Axl, PD-L1, and PD-L2 by RT-PCR and flow cytometry. Treatment of the cells with 10, 20, or 50 µg/mL of ICA significantly suppressed the relative mRNA expression and the protein surface expression of Axl, PD-L1, and PD-L2 compared to saline. Furthermore, the culture of cells in the presence of 50 µg/mL of ICA significantly inhibited the relative mRNA expression of Axl and PD-L2 and the protein surface expression of PD-L1 and PD-L2 compared to cells cultured in the presence of 10 µg/mL of ICA (Figure 7A,B). These findings suggest that ICA-mediated inhibition of Axl and immune checkpoint molecules is dose-dependent.

## 4. Discussion

The present study demonstrated that the fairy chemical ICA inhibits the expression of immune checkpoint molecules and Axl in cancer cells and melanoma xenograft growth in experimental mice.

Accumulating evidence obtained from in vitro, experimental animal, and clinical studies supports the protective role of mushroom-derived compounds against cancer [22,43,47]. For example, reports are showing that extracts from the mushroom *Phellinus linteus* block the proliferation of human lung, liver, kidney, and brain cancer cells and that the ethanol extracts from the fairy-ring mushroom Marasmius oreades inhibit the growth of human breast cancer cells and human colorectal adenocarcinoma cells [48,49]. Furthermore, preclinical studies showed that dietary supplementation of the fungus *Agaricus blazei* suppresses tumor growth, and in humans, a recent meta-analysis demonstrated that higher mushroom consumption is associated with a lower risk of developing cancer [50,51]. Importantly, clinical trials with mushroom extracts or derivatives combined with conventional anticancer therapy in patients with malignant tumors improved quality of life and reduced tumor growth [44]. To date, the beneficial health effects of mushroom extracts have been attributed mostly to the presence of polysaccharides, proteins, or nucleic acids [52,53]. Whether fairy chemicals from mushrooms exert any biological effect against cancer is unknown. In the present study, we evaluated the impact of the fairy chemical ICA on the growth of a melanoma xenograft. We found that treatment with ICA in combination with cisplatin significantly reduced the tumor growth of a melanoma xenograft in mice compared to mice treated with cisplatin alone, suggesting that ICA promotes the anticancer cytotoxic activity of cisplatin. Interestingly, the tumor tissue of mice treated with ICA and cisplatin showed increased infiltration of lymphocytes (CD3^+^ and CD8^+^) compared to mice treated with cisplatin alone, suggesting the stimulatory activity of ICA on the host immune response. In addition, AHX and AOH also inhibited the mRNA and protein expression of Axl, PD-L1, and PD-L2 in melanoma cells, suggesting that these fairy chemicals may also exert favorable effects in vivo against cancer.

Inhibition of Axl, PD-L1 and PD-L2 leads to activation of the host immune response [54,55]. Axl is a member of the TAM (Tyro, Axl, Mer) family of transmembrane receptor tyrosine kinases activated after interacting with its ligands, the growth arrest-specific-6 and protein S [54]. The TAM family of receptors, including Axl, plays an important role in regulating the immune system and tissue repair process under normal conditions [54]. Activation of Axl prevents hyperresponsiveness and autoimmunity and stimulates tissue repair by promoting the epithelial-mesenchymal transition of parenchymal cells [54]. However, excessive activation of Axl exerts detrimental effects in cancer. Stimulation of the Axl receptor tyrosine kinase leads to activation of the intracellular Akt signaling pathway that promotes cell survival and growth in tumors by suppressing pro-apoptotic proteins [15,16,56]. Axl kinase activation also inhibits the immune response in the tumor microenvironment by promoting the secretion of immunosuppressive cytokines, the secretion of PD-L1 and PD-L2, the infiltration of myeloid-derived suppressor cells, and the inhibition of T cells [17,56]. The effects of Axl kinase have been linked to enhanced tumor cell dissemination, resistance to anticancer therapy, and poor prognosis in patients with malignancy [57]. In addition to Axl-mediated mechanisms, the expression of PD-L1 and PD-L2 can also be upregulated by interferon-γ through the JAK/STAT signaling pathway [58,59]. Overexpression of these immune checkpoint molecules helps cancer cells evade immune surveillance [60]. PD-L1 and PD-L2 are ligands for PD-1, an inhibitory receptor on T cells [55]. The binding of tumor cell PD-L1 or PD-L2 to PD-1 induces apoptosis of T cells [55]. The significant reduction in tumor growth and improved survival in patients with cancer treated with monoclonal neutralizing antibodies against PD-L1 or PD-1 illustrates the important role of the immune checkpoint molecules in cancer invasiveness [55]. On this basis, we speculate that the inhibitory activity of ICA on the tumor cell expression of Axl, PD-L1, and PD-L2 explains the increased T infiltration and the favorable effects of ICA in our experimental mouse melanoma. The melanoma xenograft was resistant to cisplatin, but it became responsive to the combined therapy of cisplatin and ICA. Interestingly, ICA significantly suppressed PD-L1 downregulation induced by cisplatin in melanoma cells. Therefore, it is conceivable that resistance to cisplatin is overturned by ICA-mediated activation of the immune response. However, further studies must be undertaken to clarify the intracellular signal pathways involved in the fairy chemical-mediated inhibitory activity in cancer cells.

The in vivo experimental model performed using only one cancer cell line, the lack of dose-dependent study in the in vivo cancer model, and failure to provide the intracellular signaling pathways that mediate the inhibitory activity of fairy chemicals are limitations of the present study. However, this study introduces a highly reliable CT-based method to measure tumor growth in experimental animals and provides the initial evidence on the potential application of fairy chemicals for therapeutic purposes in cancer.

## 5. Conclusions

The present study showed that in vitro, the fairy chemical ICA inhibits the expression of immune checkpoint molecules and Axl receptor tyrosine kinase in melanoma cells and that in vivo ICA improves the therapeutic response to cisplatin in mouse melanoma xenografts. The dose-dependent inhibitory activity ICA in vitro on Axl and immune checkpoint molecules suggests the possibility to achieve even better antitumor response in vivo using higher doses of the compound. The inhibitory activity of AOH and AHX in vitro indicates that the administration of a cocktail of fairy chemicals may further potentiate antitumor responses to cisplatin in vivo. Overall, these observations point to fairy chemicals IC as promising coadjuvant therapies of conventional chemotherapy in patients with cancer.

## Data Availability

All data are available from the corresponding author under reasonable request.

## Supplementary Materials

The following supporting information can be downloaded at: https://www.mdpi.com/article/10.3390/cells11030374/s1, Figure S1: ICA potentiates cisplatin activity; Table S1: Antibodies used for Western blotting and flow cytometry analysis.
