# Supplementary material for: The Fairy Chemical Imidazole-4-carboxamide Inhibits the Expression of Axl, PD-L1, and PD-L2 and Improves Response to Cisplatin in Melanoma
Source: Cells. 2022 Jan 22;11(3):374. doi: 10.3390/cells11030374 (PMC8834508; doi:10.3390/cells11030374)
Supplement: Supplementary file 1 [file cells-11-00374-s001.zip › cells-1549038-supplementary.pdf]

Supplementary Information

The fairy chemical imidazole-4-carboxamide inhibits the ex-pression of Axl, PD-L1, and PD-L2 and improves response to cisplatin in melanoma

Chisa Inoue, Taro Yasuma, Corina N. D’Alessandro-Gabazza, Masaaki Toda, Valeria Fridman D’Alessandro, Ryo Inoue, Hajime Fujimoto, Hajime Kobori, Suphachai Tharavecharak, Atsuro Takeshita, Kota Nishihama, Yuko Okano, Jing Wu, Tetsu Kobayashi, Yutaka Yano, Hirokazu Kawagishi, and Esteban C. Gabazza.

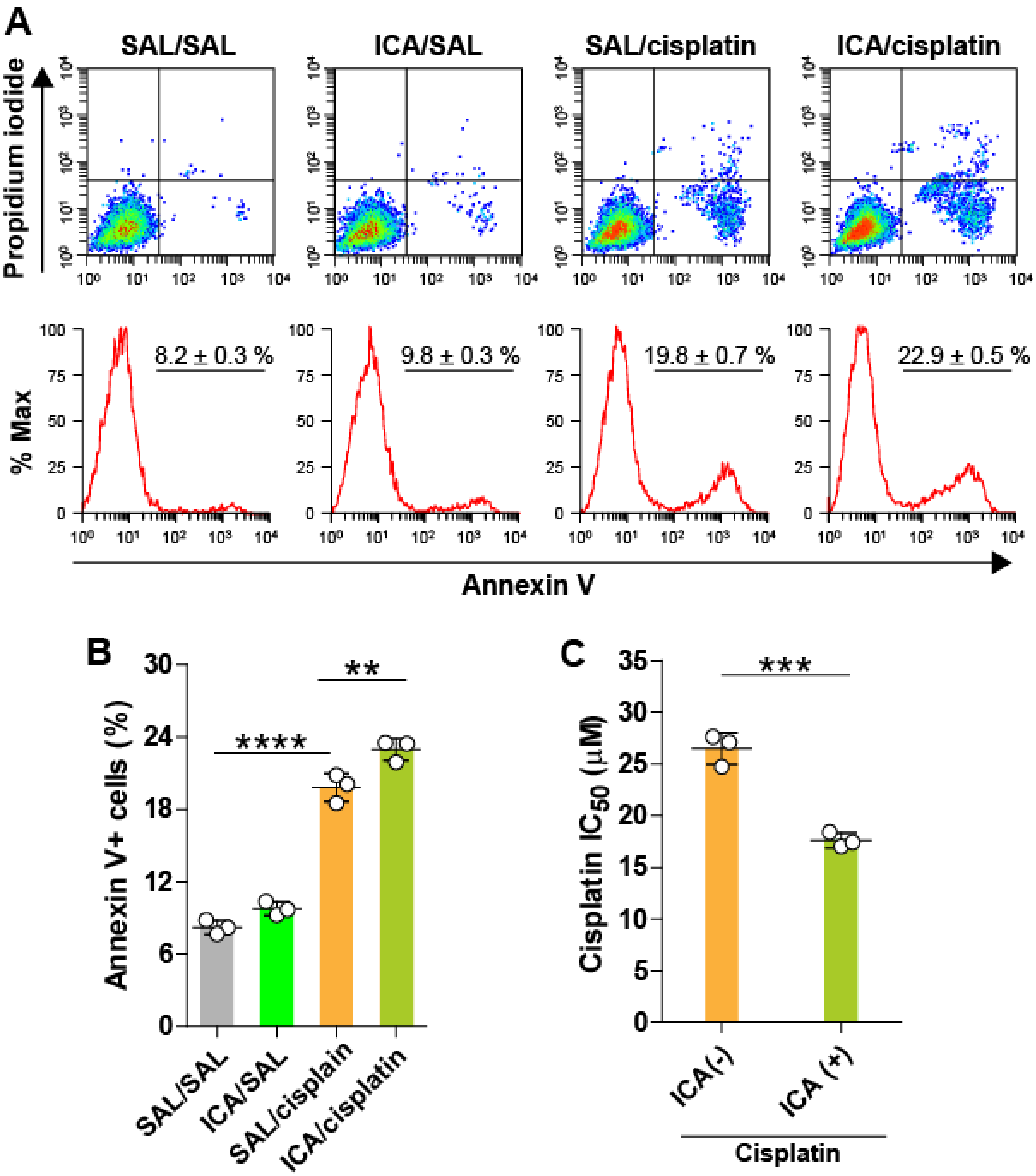

Supplementary Figure S1. ICA potentiates cisplatin activity. (A, B): B16F10 melanoma cells cultured in the presence of saline, ICA plus saline, cisplatin plus saline, or ICA plus cisplatin and apoptosis was evaluated by flow cytometry. (**C**): The 3-(4,5-dimethylthiazol-2-yl)-2,5-diphenyl-2H-tetrazolium bromide (MTT) assay to calculate the cisplatin IC50 was performed using a commercial kit. Data are expressed as the mean ± S.D. n=3 in each treatment group. Statistical analysis was performed by ANOVA with Fisher's predicted least significant difference test. **p<0.01; ***p<0.001; ****p<0.0001. ICA, imidazole-4-carboxamide; SAL, saline.

Supplementary Table S1. Antibodies used for Western blotting and flow cytometry analysis

Target	Application	Species reactivity	Manufacturers	Catalogue No	Host species & isotype
AXL	WB	human	Cell Signaling (Danvers, MA, USA)	#8661	Rabbit monoclonal antibody
AXL	WB	mouse	R&D System (Minneapolis, MN, USA)	MAB8541	Rat monoclonal antibody (IgG2a)
PD-L1	WB	human, mouse	Proteintech (Rosemont, IL, USA)	179521-1-AP	Rabbit polyclonal antibody
PD-L2	WB	human, mouse	Proteintech (Rosemont, IL, USA)	18251-1-AP	Rabbit polyclonal antibody
AXL	FC	mouse	R&D System (Minneapolis, MN, USA)	FAB8541-R	Rat monoclonal antibody (IgG2a)
PD-L1	FC	mouse	BioLegend (San Diego, CA, USA)	155404	Rat monoclonal antibody (IgG2a)
PD-L2	FC	mouse	BioLegend (San Diego, CA, USA)	107205	Rat monoclonal antibody (IgG2a)
Phosphatidylserine	FC		FITC-Annexin V, BioLegend (San Diego, CA, USA)	640906	
DNA	FC		Propidium iodide, BioLegend (San Diego, CA, USA)	556463	

PD-L1, programmed death-ligand 1; PD-L2, programmed death-ligand 2; WB, Western blotting; FC, flow cytometry; FITC, fluorescein isothiocyanate.
